# Investigation of potential pathogenicity of *Willaertia magna* by investigating the transfer of bacteria pathogenicity genes into its genome

**DOI:** 10.1038/s41598-019-54580-6

**Published:** 2019-12-04

**Authors:** Issam Hasni, Nisrine Chelkha, Emeline Baptiste, Mouh Rayane Mameri, Joel Lachuer, Fabrice Plasson, Philippe Colson, Bernard La Scola

**Affiliations:** 1 Aix-Marseille Université UM63, Institut de Recherche pour le Développement IRD 198, Assistance Publique – Hôpitaux de Marseille (AP-HM), Microbes, Evolution, Phylogeny and Infection (MEΦI), Institut Hospitalo-Universitaire (IHU) - Méditerranée Infection, Marseille, France; 2 Amoéba, Chassieu, France; 3 ProfileXpert/Viroscan3D, UCBL UMS 3453 CNRS – US7 INSERM, Lyon, France; 4 Inserm U1052, CNRS UMR5286, Centre de Recherche en Cancérologie de Lyon, Lyon, France; 5 Université de Lyon, Lyon, France

**Keywords:** Computational biology and bioinformatics, Phylogenetics

## Abstract

*Willaertia magna* c2c maky is a thermophilic amoeba closely related to the genus *Naegleria*. This free-living amoeba has the ability to eliminate *Legionella pneumophila*, which is an amoeba-resisting bacterium living in an aquatic environment. To prevent the proliferation of *L. pneumophila* in cooling towers, the use of *W. magna* as natural biocide has been proposed. To provide a better understanding of the *W. magna* genome, whole-genome sequencing was performed through the study of virulence factors and lateral gene transfers. This amoeba harbors a genome of 36.5 megabases with 18,519 predicted genes. BLASTp analyses reported protein homology between 136 *W. magna* sequences and amoeba-resistant microorganisms. Horizontal gene transfers were observed based on the basis of the phylogenetic reconstruction hypothesis. We detected 15 homologs of *N. fowleri* genes related to virulence, although these latter were also found in the genome of *N. gruberi*, which is a non-pathogenic amoeba. Furthermore, the cytotoxicity test performed on human cells supports the hypothesis that the strain c2c maky is a non-pathogenic amoeba. This work explores the genomic repertory for the first draft genome of genus *Willaertia* and provides genomic data for further comparative studies on virulence of related pathogenic amoeba, *N. fowleri*.

## Introduction

Heterolobosea is a rich class of species of heterotrophs, almost all free-living, containing pathogenic (e.g. *Naegleria fowleri*) and non-pathogenic (e.g. *Willaertia magna*) amoebae^1–5^. Among the nearly 150 species described, only three had their genomes sequenced: *Naegleria gruberi*, *Naegleria fowleri* and *Naegleria lovaniensis*^6–8^. *W. magna* belongs to the family *Vahlkampfiidae* and has been described for the first time by De Jonckheere *et al*. in 1984^3^. The trophozoite form is large and varies from 50 to 100 μm in length. The flagellate form typically has four flagella, and the cysts are 18 to 21 μm in size. It is thermophilic as it can grow in high temperatures, up to 44 °C^3,9^. Amoebae are natural predator of microorganisms in the environment but can also serve as vector for microorganisms that have evolved to resist phagocytic lysis after amoebal internalization^10,11^. These organisms are named amoeba resisting microorganisms (ARMs). The interactions between ARMs and amoebas have been demonstrated to promote gene exchanges during evolution^12–15^. Beside ARMs, free living amoeba, especially *Acanthamoeba* spp., have been also demonstrated to support the growth of giant viruses^16^. Some of these ARMs are human pathogens, especially *Legionella pneumophila*^17^. *L. pneumophila* is naturally present in aquatic environments and man-made water systems, such as cooling towers. The proliferation and dissemination of *L. pneumophila* in water of cooling towers are related to legionellosis outbreaks, a respiratory disease transmitted by inhalation or by breathing contaminated aerosol^18,19^. The *L. pneumophila* eradication in aquatic system is difficult as it can survive in biofilms and amoebae^20^. Indeed, amoebae provide *L. pneumophila* a protection against adverse environmental conditions and disinfectants. Further, the replication of *L. pneumophila* within this amoebal host has been reported to allow the dissemination of pathogen in the environment^21,22^. In contrast with other free-living amoebas, such as *Acanthamoeba* spp. or *Vermamoeba vermiformis*, the strain *W. magna* c2c maky has been specifically demonstrated to eliminate strains of *L. pneumophila* after phagocytosis^23^. Based on this property, the Amoeba® company (http://www.amoeba-biocide.com/fr) has proposed *W. magna* as a natural biocide to control *L. pneumophila* in cooling towers while reducing chemical biocides commonly used in water treatment. To expand our knowledge on *W. magna* c2c maky, we conducted genomic analysis on *W. magna* c2c maky strain and performed comparative genomic analyses with the genomes of other amoeba from the same family, including the human pathogen *N. fowleri*, in the search for genes related to the virulence and exchange of putative sequences with amoeba-resistant microorganisms and giant viruses. Finally, we performed a cytotoxicity test on human cells to confirm the data obtained by bioinformatics analysis.

## Results

### Main genomic features

The genome length of *W. magna* is 36.5 megabases (Mb), encompassing 4,505 scaffolds, which is smaller than the genome of *N. gruberi* (41 Mb), but larger than the genome of *N. fowleri* (30 Mb) and *N. lovaniensis* (30.8 Mb) (Table 1). The guanine-cytosine (GC) content of the genome of *W. magna* is 25%, significantly lower than that of the closely related amoebae of the genus *Naegleria* of 36% (Table 2). It is rather comparable to the distant *Dictyostelium discoideum* and *Entamoeba histolytica* with 22% and 24% of GC-content respectively. Moreover, the analysis of assembly quality control based on taxon-annotated GC coverage had reported that the assembly components from *W. magna* c2c genome had similar proportion of GC%, and the same coverage in the raw data (Supplementary Fig. S1). These results are predictive of low GC content, which is probably not related to contamination. The phylogenetic analysis based on partial 18S rRNA gene showed that *W. magna* strains c2c maky is most closely related to *Naegleria* species (Supplementary Fig. S2).

**Table 1:** Summary of the *W. magna* c2c genome.

Parameter	Number
Haploid genome size (bp)	36,587,372
Sequence contigs (n)	4,505
GC-content (%)	24.8
Maximal scaffold size (bp)	224,412
Minimal scaffold size (bp)	970
Average scaffold size	8121
N25	37,064
N50	16,266
N75	6,898

bp, base pairs; N50, 50% of the genome assembly is as contigs larger than this size; N25, 25% of the genome assembly is as contigs larger than this size.

**Table 2:** Comparison of the main genomic features of several amoebas.

Organisms	Genome size (Mb)	Predicted proteins	Annotated proteins	G + C%
*Willaertia magna* c2c maky	37	18,519	13,571	25%
*Naegleria fowleri* ATCC 30863	30	17,252	16,021	35%
*Naegleria gruberi* NEG-M	41	15,727	9,090	33%
*Naegleria lovaniensis* ATCC 30569	31	15,195	13,005	37%
*Dictyostelium discoideum* AX4	34	13,541	8,422	22%
*Entamoeba histolytica* strain HM-1: IMSS	21	8201	4076	24%

Source of data: *Naegleria fowleri*^7^, *Naegleria gruberi*^6^*, Naegleria lovaniensis*^8^, *Dictyostelium dis*coideum^71^ and Entamoeba histolytica^72^.

### Functional annotation

**Figure 1:**
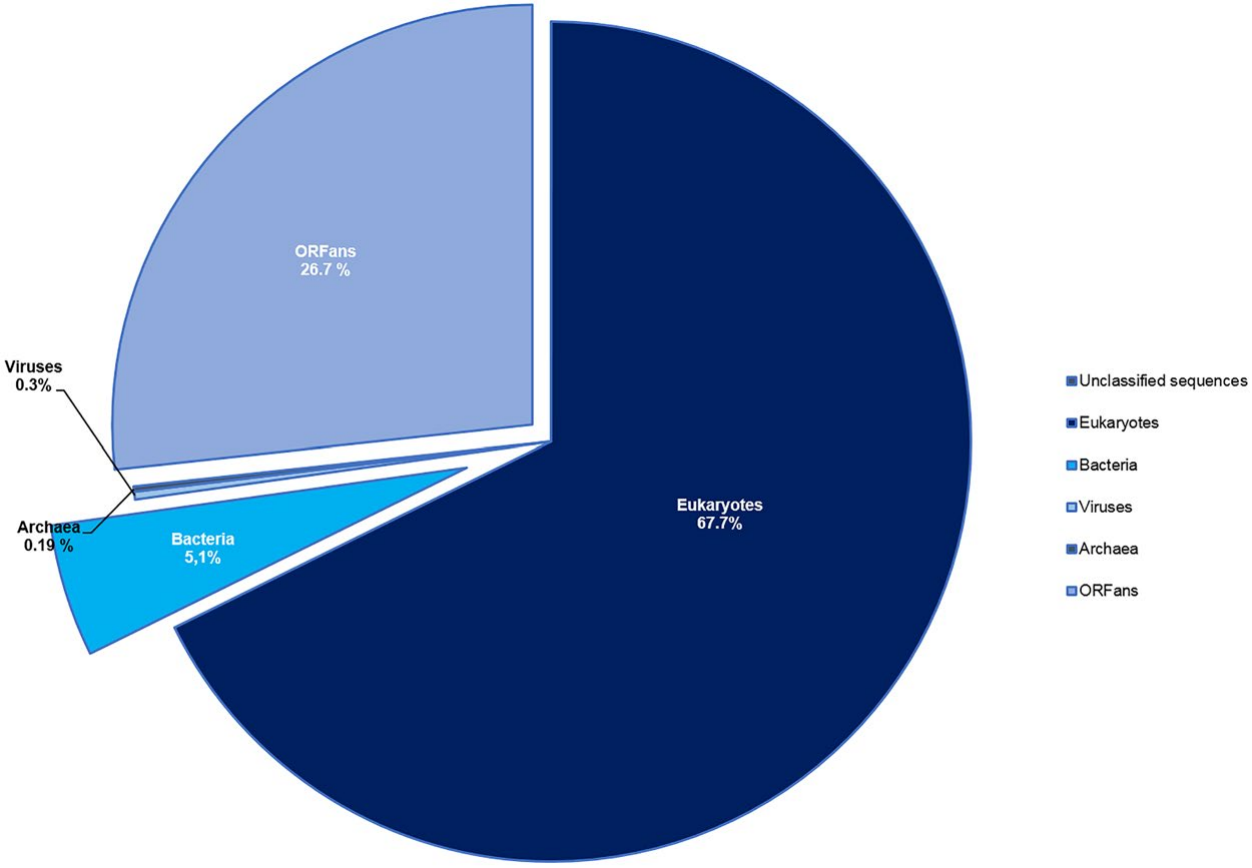
Taxonomic distribution of BLASTp best hits. *W. magna* shared 67.7% of the predicted proteins with eukaryotes, 5.1% with bacteria, 0.19% with archaea and 0.3% with viruses. 26,7% of *W. magna* protein sequences show no similarity with known proteins in NR database.

**Figure 2:**
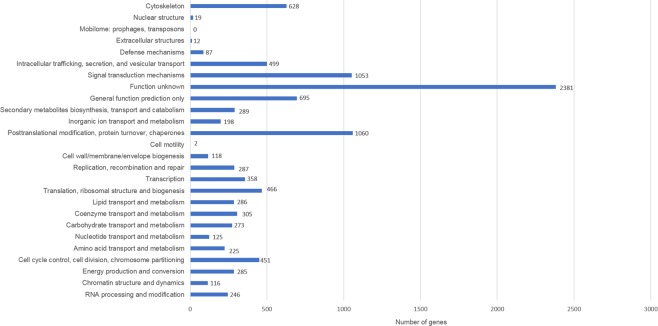
Number of genes associated with the general COG functional categories.

BLASTp comparison against nr database allowed to assign a putative function at 13,571 protein sequences (73.3%) and 4,948 are ORFans (26.7%). The taxonomical distribution of *W. magna* coding sequences showed that 67.7% of the annotated proteins are shared with eukaryotes, 5.1% with bacteria, 0.19% with archaea, 0.3% with viruses and 0.01% unclassified sequences (Fig. 1). The mapping of protein sequences against the COG database made it possible to annotate the function of 9,164 genes (49.5% of the genome) (Fig. 2). The analysis revealed a high number of genes involved in post-translational modifications, signal transduction mechanisms and cytoskeletons, equal to 1,060, 1,053 and 628, respectively. The proportion of genes related to cytoskeleton is greater for *W. magna* (3.4%) than for *Naegleria* species, including *N. fowleri* (2.3%), *N. gruberi* (2.3%) and *N. lovaniensis* (2.3%) (Supplementary Fig. S3), which could be related to the high mobility of *W. magna*. In addition to the cytoskeleton-related genes, the analysis of the genome content provided information about the specific genes involved in the mechanism of flagellar formation that allows *W. magna* to move in the aquatic environment (Supplementary Table 1). The comparison of protein sequences with the KEGG database (http://www.kegg.jp/kegg/kegg1.html.) reported 4,708 (25.5% of genome) significant matched. Among them, 1,780 genes are assigned to metabolic pathways and classified into 11 subcategories (Supplementary Fig. S4). The genes involved in carbohydrate pathways (381 genes) are the largest represented, followed by amino acid (299 genes) and lipid (266 genes) pathways. Several genes coding for enzymes involved in the biosynthesis of natural products were found within the genome of *W. magna*. Indeed, we identified genes (isopentenyl-diphosphate Delta-isomerase, farnesyl-diphosphate diphosphomevalonate decarboxylase and farnesyltransferase) involved in the biosynthesis of terpene precursors and squalene. These findings suggest that the amoeba could synthesize terpene compounds that constitute a large structural group of natural products with a wide range of biological properties^24^ (Supplementary Table 1 and Supplementary Figs. S5, S6 and S7). The metabolic pathway investigation showed the presence of genes involved in the biosynthesis of secondary metabolites and antibiotics, we notably reported the presence of an enzyme involved in the biosynthesis of penicillin (K01434: penicillin amidase) (Supplementary Table 1 and Supplementary Fig. S8). Furthermore, we found multiple-copies of genes coding for beta-lactamase responsible for resistance to beta-lactam antibiotics (Supplementary Table 1). Although *W. magna* is grown in axenic medium, the genome analysis reported a set of genes involved in the degradation of bacterial peptidoglycan and polysaccharide storages (Supplementary Table 1). In addition, the identification of esterase, phospholipases and lipase, which are related to the phago-lysosomal system, suggests that *W. magna* still has the ability to digest the bacteria (Supplementary Table 1). Metabolism study of *N. gruberi* showed that the amoeba grown in axenic medium also retained the set of genes involved in the bacterial degradation^25^.

### Genomic comparison with other amoebae from the family *Vahlkampfiidae*

**Figure 3:**
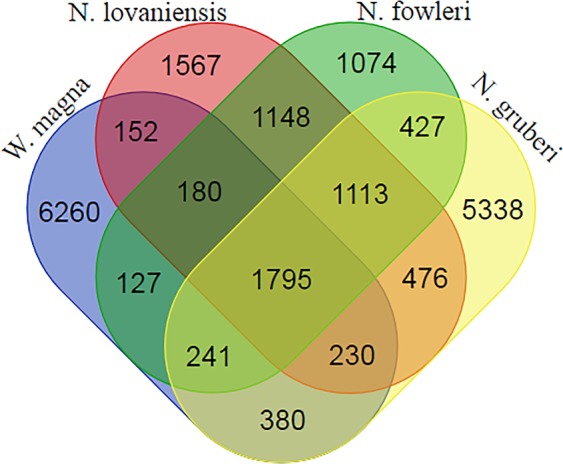
Venn diagram comparison of ortholog gene number obtained belonging to the predicted proteins of *W. magna*, *N. gruberi*, *N. fowleri* and *N. lovaniensis*.

*W. magna*, *N. fowleri*, *N. gruberi* and *N. lovaniensis* exhibited a pangenome and a core genome of 20,554 and 1,795 genes, respectively (Fig. 3). The core genome therefore represented 8.7% of the pangenome. We found 121 protein sequences that formed 57 clusters of paralogous genes comprising small numbers of genes ranging between two and four. Based on the comparison of 4 strains sequences, the functional annotation of the core genome reported a high number of genes related to metabolism (407 genes), unknown function (353 genes) and posttranslational modification (208 genes) (Supplementary Fig. S9). Regarding the genome sequence of *W. magna*, the unique genes are rather related to unknown function (805 genes), signal transduction (334 genes) and cytoskeleton mechanism (210 genes) (Supplementary Fig. S10). Comparisons with other *Vahlkampfiidae* member genomes showed that *W. magna* had a majority of orthologs (n = 2,754) in *N. gruberi*, a non-pathogenic amoeba. Indeed, *W. magna* had 14.3%, 12.7% and 12.6% of orthologs shared with *N. gruberi*, *N. lovaniensis* and *N. fowleri*, respectively. The phylogenetic tree based on the presence and absence of homologous genes showed that genes could be shared at different rates by horizontal transfer between *W. magna* and each *Naegleria* species (Supplementary Fig. S11).

### Possible horizontal gene transfers between *Willaertia magna* and Amoeba-Resistant Microorganisms

**Figure 4:**
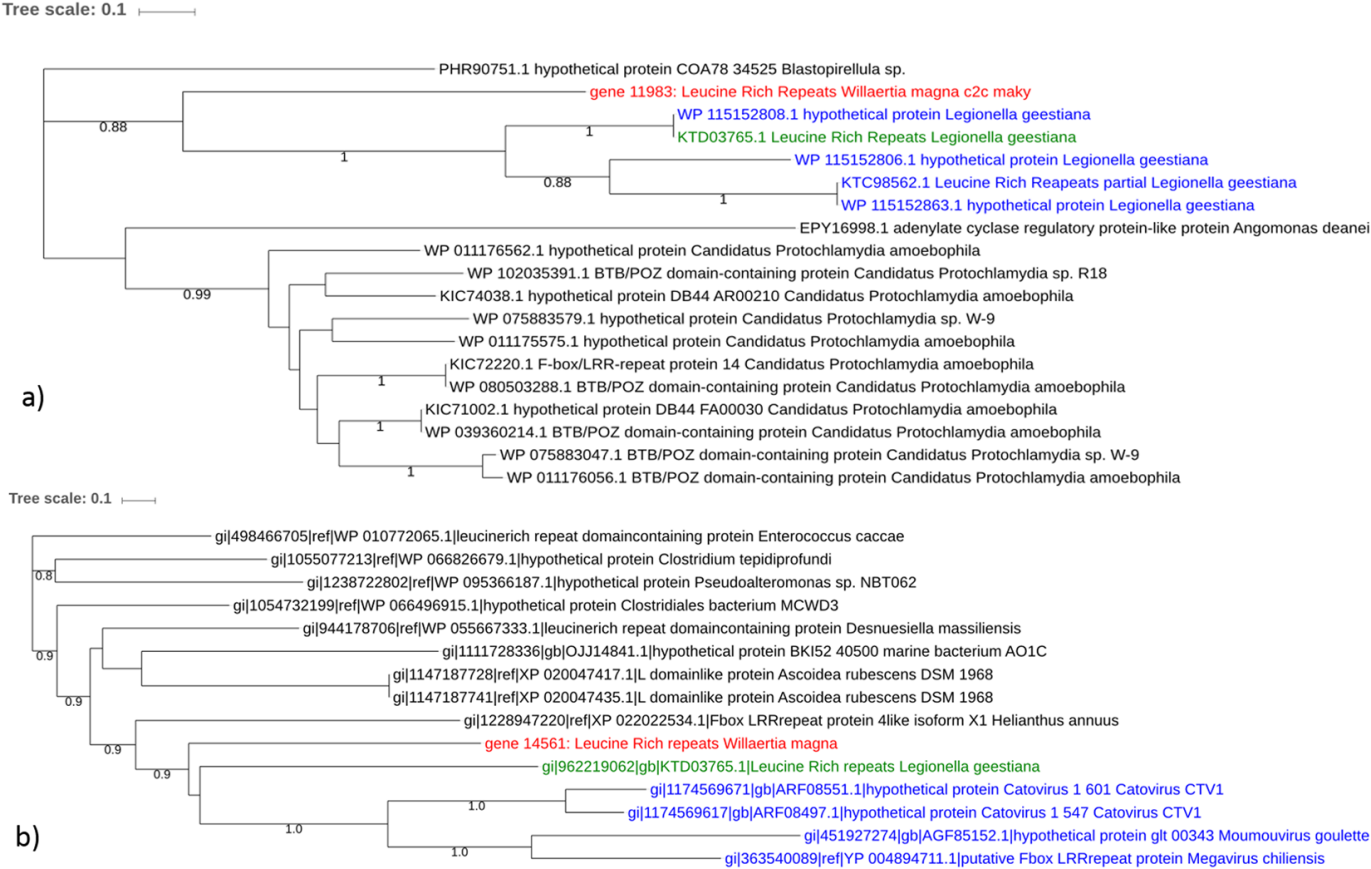
Representation of horizontal transfer analysis. Phylogenetic trees for two *W. magna* c2c maky proteins of putative ARB origin. The trees were constructed using maximum-likelihood method based on the leucine rich repeat sequence of *W. magna*. The trees were performed with 18 (**a**) and 14 (**b**) homologous sequences of *W. magna* retrieved by BLASTp on NCBI. In red: *W. magna* c2c maky leucine rich repeat-containing protein (**a**) gene 14,561; (**b**) gene 11,963); in green: the ARB (*legionella geestiana*) homologs; in blue: homologs from *legionella geestiana* (**a**) or homologs from giant viruses (**b**); in black: homologs from other organisms.

A total of 136 genes were found to have best-hits in nr database with amoeba-resistant microorganisms (ARMs), including amoeba-resistant bacteria (ARBs), amoeba endosymbionts and giant viruses (Supplementary Table S2). These genes are mostly general and of unknown functions (n = 94, 68%), followed by post-translational modifications (n = 19, 14%) and molecule transport and metabolism related genes (n = 7, 5%) (Supplementary Fig. S12). The majority of these 136 genes (87 genes; 64%) are shared with bacteria that can survive within amoebae. As a matter of fact, the obligate intracellular bacteria belonging to the *Chlamydiae* phylum were the ARBs with which *W. magna* exhibited the highest number of best hits (50 genes; 36.5%). Multiple genes best matching with amoeba endosymbionts (10 genes; 7.3%*)* from various clades were also found within the *W. magna* c2c maky genome (Supplementary Table S2)*. W. magna* shared 10 genes (12.5%*)* with bacterial species isolated in amoebae from environmental samples, such as *Mycobacterium* sp., *Pseudomonas* sp., *Acinetobacter* sp^26^. or with bacteria able to survive *in vitro* within amoebae, such as *Rickettsia bellii*^27^. Finally, we identified 17 *Legionella* sp. genes potentially exchanged with *W. magna* (12.5%). These results suggest a substantial amount of horizontal gene transfers between *W. magna* and ARBs (Supplementary Fig. S13). Among the 52 *W. magna* genes related to viral sequences, 49 had a best BLASTp hit with giant virus genes. The majority of these viral sequences (48 genes) belong to *Mimiviridae* members, including genes of mimiviruses of lineages A-C (48%) and of klosneuviruses (50%). The majority (n = 38) of *W. magna* genes best matching with viral sequences encode hypothetical proteins. Other genes of this type encode leucine rich repeat-containing proteins (*Lrrc* (n = 3)) and F-box domain-containing proteins (*Fbxw* (n = 2)) (Supplementary Table S2). In addition, *W. magna* c2c maky genes best matched with a gene encoding a DNA-directed RNA Pol II C-terminal-like phosphatase from *Pithovirus sibericum*, which is a protein involved in the translation pathway (Supplementary Table S2). The phylogenetic trees based on *W. magna* best hits with ARMs were performed to evidence the lateral transfers of genes. Among the 136 *W. magna* best hits with the ARMs, phylogenetic analyses revealed that the lateral gene transfers were confirmed for 38 (27.7%) protein sequences, including 18 and 20 that had a best hit with ARBs and giant viruses, respectively. The phylogeny reconstruction, showed in Fig. 4a, seems to indicate that the closest homolog to the *W. magna* gene identified so far was found in *Legionella geestiana*, and suggests that a gene encoding a leucine rich repeat-containing protein (*Lrrc*) was transferred between *W. magna* and *L. geestiana*. A phylogenic tree based on a *Lrrc* protein of *W. magna* was clustered with homologs from *L. geestiana* and giant viruses (Fig. 4b). According to this phylogeny, we can hypothesize that the gene sequence was transferred between *W. magna, L. geestiana*, and giant viruses within *W. magna*.

### Analysis of potential virulence related genes

**Figure 5:**
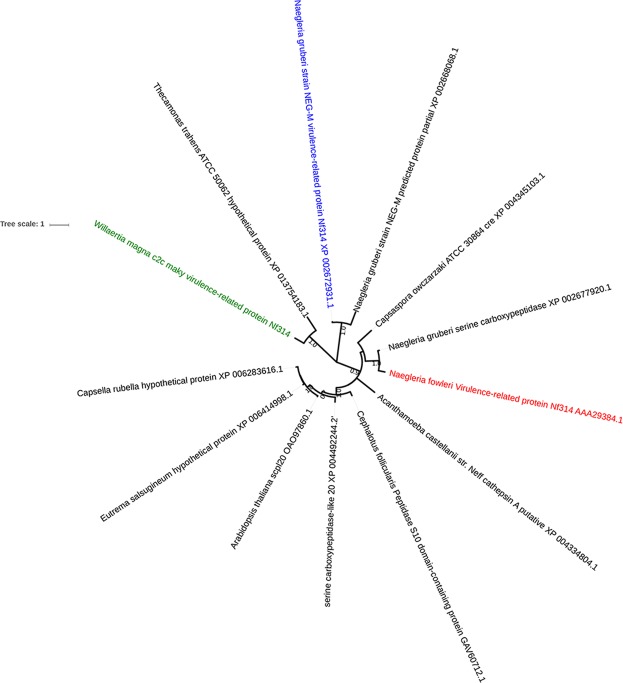
Phylogenetic analysis of *W. magna* c2c maky *Nf314* protein. The phylogenetic tree is based on protein *Nf314* from *W. magna* c2c and other eukaryotic organisms including amoebas. *Nf314* protein sequences of *W. magna* are not clustered with this homolog in *N. fowleri*. The protein *Nf314* of *W. magna* c2c is indicated in green; that of non-pathogenic amoeba in blue and that of pathogenic amoeba (*N. fowleri*) in red.

We detected 15 genes with known function shared with bacteria, mostly living in the environment and identified as potentially pathogenic for humans^28–35^ (Supplementary Table S3). The comparison of these sequences with those of a virulence and pathogenic factor database did not report any significant similarity with the bacterial virulence factors described. The analysis of the 67 genes shared with the pathogenic amoeba *N. fowleri* showed that 15 genes had as best BLASTp hits genes associated with *N. fowleri* virulence^7^, encompassing genes encoding heat shock protein 70 (*Hsp70* (n = 2)), Nf314 protein (n = 1), actin protein (n = 2), and *Mp2CL5* membrane protein (n = 10) only detected in *N. fowleri* (Supplementary Table S4). A *Nf314* gene identified in the *W. magna* genome had as best hit a *N. gruberi* gene but its homolog in *N. fowleri* encodes a virulence factor. However, BLASTp analyses found that all these *N. fowleri* genes have as homolog genes of *N*. *gruberi*, a non-pathogenic amoeba (Supplementary Table S5). Phylogenetic reconstructions showed that the *N. fowleri Mp2CL5* gene was clustered with homologous sequences from non-pathogenic amoebas *N. gruberi* and *W. magna* (Supplementary Fig. S14), and that the *Hsp70*, actin, *Nf314* genes in *W. magna* c2c maky were apart from their corresponding homologous sequences in *N. fowleri* (Fig. 5; Supplementary Fig. S14). It is therefore unlikely that these proteins play a putative role as a human virulence factor in *W. magna* c2c maky.

### Cytotoxicity assay

**Figure 6:**
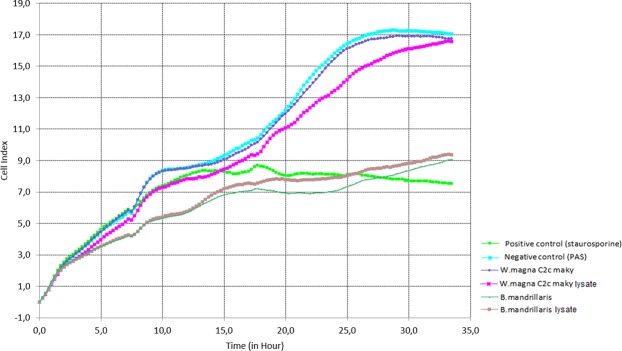
Dynamic monitoring of the amoebae cytotoxicity effects on ECs using iCELLigence real time cell analysis system. Each curve represents the cell index of the ECs in co-incubation with different effectors. In blue light: PAS, in green light: staurosporine, in pink dark: *W. magna* lysate, in blue dark: *W. magna* trophozoites, in brown: *B. mandrillaris* lysate and in green dark: *B. mandrillaris* trophozoites. The decrease in cell index is correlated with detachment of the ECs and a cytopathogenic effect of the effector on ECs. *B. mandrillaris* lysate and trophozoite had a similar effect on ECs that positive control (decreasing of the cell index), while *W. magna* lysate and trophozoite had a similar effect on ECs as the negative control (increasing of the cell index).

After 30 hours of culture, the cell index of the positive control (staurosporine) decreased to 7.7 as a mean value of all registered values, while the negative control (endothelial human cells with page’s modified Neff’s amoeba saline medium) had a cell index that reached 17.2 at its maximum registered value (Fig. 6). This cell index showed good adhesion, and proliferation regarding the positive control. *W. magna* and its lysate showed a cell index of 16,9 to 16,5 respectively (Fig. 6). For these assays, the proliferation is similarly to negative control and no significant difference was observed between the values of *W. magna* cell index and the negative control cell index. In contrast, endothelial cells (ECs) with *Balamuthia mandrillaris* trophozoite form and its lysate showed a significant decrease in the cell index with values of 8.4 and 8.8 respectively at the end of the registered values (Fig. 6). So, we can suggest that *W. magna c2c maky* and its lysate showed no effect nor cytotoxicity on ECs, whereas *B. mandrillaris* cytotoxicity prevented ECs growth.

## Discussion

*W. magna* c2c maky is the fourth draft genome available for an amoeba from the family *Vahlkampfiidae* and provides new insights about the gene repertoire of the amoebae belonging to the *Heterolobosea*. The analytical data provided present features on the main characteristics of the unicellular eukaryotic with a significant genome of 36.5 Mb including 4,505 scaffolds. Its GC-content is extremely rich and about 10% lower than the GC-content of *Naegleria* species^6–8^. This particular GC-content could influence amino acid composition and codon usage, as described for the *Dictyostelium discoideum* genome^36^. The draft genome sequence of *W. magna* is composed of gene sequences from diverse putative origins. However, *W. magna* had shared a high number of best hits with amoebas of the same family (57.8% of the genome). The genome study revealed the presence of several genes involved in bacterial degradation and the biosynthesis of natural products such as terpenes and secondary metabolites. In their natural environment, amoebas are in constant competition with other organisms for food^36,37^. In addition, amoebae must cope with predation and toxins to survive. So, *W. magna* expanded a set of protein classes to fight and protect themselves against the interactions with others amoebas, fungi and bacteria also present in this ecosystem^36,37^.

Amoebae are ecological niches for microorganisms that live in symbiosis, allowing sequence exchanges, not only between intra-amoebal microorganisms, but also between these microorganisms and amoebae^12,14,38^. For instance, there is previous evidence of horizontal sequence transfer between *Legionella drancourtii* and *A. castellani* Neff of a gene encoding a malate synthase^12^. In the present work, BLASTp and phylogenetic analyses indicated horizontal gene transfers between *W. magna and* ARBs, including genes of obligate intracellular bacteria, such as *Chlamydia* and *Rickettsiales* endosymbionts, diverse *Legionella* species and *Acinetobacter lowfii*. We also found 49 genes involved in sequence transfers between giant amoeba viruses (mimiviruses in all but one case) and *W. magna*. The occurrence of homologs of *W. magna* genes in giant viruses suggests that this amoeba might represent an ancient host for these giant viruses. The proportion of such giant viral sequences in *W. magna* (0.3%) is lower than that inferred for *Acanthamoeba castellani* Neff, for which 76 (0.5%) and 267 genes (1.2%) were involved in lateral transfers with giant viruses as reported by Clarke *et al*. (2013) and Maumus *et al*.^13,14^, respectively (p < 0.001). This proportion was greater than that described in *Acanthamoeba polyphaga* (0.1%), considering that the number of ORFs detected in its draft genome sequence was much higher^15^. This low number of *W. magna* genes best matching with giant viral genes could be explained by the fact that no giant viruses have been currently isolated in amoebae of the genus *Willaertia*, or other phylogenetically related amoebae such as *Naegleria*. Therefore, some of the *W. magna* ORFans could be giant virus sequences that have not been isolated so far and their close relatives or ancestors. In the majority of cases, a sense for the gene transfers could not be proposed based on the topology of the tree. As *W. magna* c2c maky is the only genome of the genus *Willaertia* available, the lack of homologous sequences from other *Willaertia* species might have complicated the interpretation of the phylogenetic trees. Nevertheless, according to some phylogenetic reconstructions, the most parsimonious evolutionary scenario was a sequence transfer from *W. magna* to ARMs, while in other cases, the most parsimonious evolutionary scenario was a sequence transfer in the opposite sense, from ARMs to *W. magna*. Besides, a phylogeny reconstruction has shown cluster composed of giant virus sequences, ARBs and *W. magna*, confirming the paradigm that amoebas grazing on microorganisms constitute a biological niche promoting genetic exchanges. Finally, a large part of sequence exchanges between *W. magna* and ARMs involved hypothetical proteins. However, these putative proteins could play an important role in the adaptation and evolution of these organisms. The co-existence of pathogenic bacteria and amoebas in the aquatic environment can facilitate the horizontal gene transfers between these microorganisms. So, we investigated the presence of homologous genes potentially belonging to human pathogenic bacteria within the *W. magna* genome. The comparative analysis revealed 15 best BLASTp hits with genes from virulent bacteria. Furthermore, we found 1 best hit with a gene from *Aeromonas hydrophila*, an enteropathogenic organism that is an inhabitant of the aquatic environment and demonstrated the ability to survive within *Acanthamoeba castellanii* trophozoites and cysts^39^. However, the genomic analysis reported that none of these genes with homologs in virulent bacteria and found in the *W. magna* genome were related to the pathogenicity. *W. magna* has never been reported as the causal agent of infections in humans, as for the case of *Acanthamoeba* species that have been involved in keratitis and encephalitis, or *B. mandrillaris* that has been associated with encephalitis^40,41^. We found that *W. magna* is phylogenetically close to *Naegleria* species, including *N. fowleri*, an opportunistic pathogen that caused primary amoebic meningoencephalitis, a rare but deadly disease in humans^42,43^. To improve knowledge on *N. fowleri* pathogenicity, several proteins, potentially involved in virulence, have been identified^7^.In this work, we analyzed the potential virulence gene of *N. fowleri* shared with *W. magna* c2c maky. Ju Song *et al*. (2008) demonstrated that *N. fowleri* heat shock proteins 70 (*Hsp*70), abundant molecular chaperones, are implicated in the adaptive responses for survival and pathogenicity mechanisms^44^. Actin proteins related to cell mobility and division and *Nf314* serine carboxypeptidase have also been proposed to be potential *N. fowleri* virulence factors since these proteins were over-expressed in highly virulent *N. fowleri* trophozoites^7,45^. Another protein named *Mp2CL5* was identified as potentially involved in the phagocytosis and cell proliferation^46^. This membrane protein has only been isolated in *N. fowleri* and has been up-regulated in highly pathogenic *N. fowleri*^47^. These four proteins have homologous in the *W. magna* draft genome. Nevertheless, genes coding for actin, *Hsp70* and *Nf314* also exist in the genome of *N. gruberi*, a non-pathogenic species. In addition, a homolog of the *N. fowleri Mp2CL5* protein might be present in *N. gruberi*. Moreover, phylogenetic analyses show that virulence related genes of *N. fowleri* and present in *W. magna* are clustered with proteins from non-pathogenic amoebas. This finding does not explain the specific virulence of *N. fowleri* in humans, despite their implication in pathogenicity. It might be interesting to perform a transcriptomic analysis to observe whether these virulence related genes in *N. fowleri* are expressed or not in *W. magna*.

Several other studies have demonstrated that virulence factors are present in both pathogenic and non-pathogenic amoeba^48,49^. Moreover, homologs of genes from virulence gene database were not found in the draft genome of *W. magna* c2c maky. This is the case of matrix metalloproteinases that are essential enzymes required in *N. fowleri* during the process of degradation of the extracellular matrix^50^. The presence of *N. fowleri* proteins annotated as potential virulence factors in the genomes of other amoebas is not sufficient evidence that these amoebas can be human pathogens.

The in-silico study of the *W. magna* c2c maky pathogenicity was not sufficient to conclude on the non-virulence of the strain c2c maky. We therefore performed a cytotoxicity test on human cells with *W. magna* c2c maky and a virulent amoeba strain (*B. mandrillaris*) which showed in previous studies a cytotoxic effect on human cells^51^. The result of the cytotoxicity assay revealed that *W. magna* c2c maky cells and *W. magna* c2c maky lysate probably have no cytotoxicity effect on ECs, whereas *B. mandrillaris* has degraded the ECs. This lack of cytotoxic effect suggests that no toxins are produced by *W. magna* c2c maky that could harm the development of human endothelial cells.

These results are fully consistent with the Matin study which has shown that *B. mandrillaris* is able to produce metalloproteases capable to destroy extracellular matrix by degradation of collagen I and III, elastin, plasminogen, as well as other substrates, such as casein and gelatin^51^. The production of *B. mandrillaris* protease is essential for the attachment and the invasion in human cells leading to the granulomatous amoebic encephalitis. Furthermore, our findings are in congruent with *in-vivo* observations that reported the non-pathogenicity of the *W. magna* species revealed by the non-infection of mice after the injection of *W. magna*^3,4^.

Therefore, the sequencing and genomic analysis of *W. magna* c2c maky provides additional information on the unexplored world of the amoebas of the *Vahlkampfiidae* family. The genome is composed of genes from various origins. Furthermore, the study of interaction with amoeba-resisting microorganisms reported a potential exchange of sequences. Moreover, the survey on virulence and pathogenicity demonstrated that *W. magna* is a non-pathogenic amoeba, unlike *N. fowleri*, which is a closely related organism of the same family.

## Materials and Methods

### *W. magna* c2c maky production

*W. magna* c2c maky cells (ATCC PTA-7824) were grown at 30 °C in 175 cm² culture flasks (Thermo Fisher Scientific, Illkirch, France) containing 75 mL of SCGYEM medium^52^. After 72 hours of incubation, the amoebae were detached from culture flask and harvested by centrifugation at 1000 g for10 minutes followed by three steps of washing using Page’s modified Neff’s Amoeba Saline medium (2 mM NaCl, 16 μM MgSO4, 27.2 μM CaCl2, 1 mM Na2HPO4, 1 mM KH2PO4). Amoeba quantification was performed using a KOVA® slide cell counting chamber.

### Nuclear DNA extraction and sequencing

DNA of *W. magna* c2c maky was extracted with 1 volume of phenol/chloroform/isoamyl alcohol (50:49:1) (Merck KGaA). After 5 minutes of centrifugation at 13 000 rpm, the aqueous phase was recovered. This DNA extraction step was repeated twice. The DNA was precipitated with the addition of 2 volumes of ethanol (Merck KGaA) and gently stirred by hand. The suspension was incubated overnight at −20 °C, centrifuged at 13 000 rpm for 30 minutes at 4 °C, the supernatant was removed and the DNA solution dried in the air. DNA was dissolved in 0.5 ml in TE buffer (10 mM Tris, 0.1 mM EDTA). Then, gDNA was quantified using a QuantiFluor® dsDNA System kit (Promega, Charbonnières, France) to 113 ng/µl and qualified on agarose gel 1%. To perform sequencing, 2 µg of genomic DNA was sonicated using the Bioruptor Next Gen sonication device (Diagenode SA, Belgium) and DNA libraries were generated using the NEXTflex PCR-Free DNA-seq library prep kit (Bioo Scientific Corporation, Austin, United States). Library size was assessed on BIOAnalyser 21000 from Agilent using High Sensitivity DNA chip 2100 (Agilent Technologies Inc., Santa Clara, CA, United States) and quantified using QuantiFluor® dsDNA System kit (Promega, Charbonnières, France) and by specific qPCR. Library size was about 400 bp, which is compatible with cluster generation. Library was paired-end sequenced (2 × 150 bp) with a Mid Output flow cell on the NextSeq. 500 sequencer (Illumina, Inc., San Diego, CA, United States) corresponding to a depth of X1000. DNA sample sequencing generated 153.86 million of raw read pairs (307.72 million raw reads), 91.5% of which met the Illumina filtering criteria. In order to verify the absence of contamination in the DNA sample, a random subset of one million reads has been aligned using the FastQ Screen tool^53^ against a local database containing the main sources of contamination.

### RNA extraction and sequencing

RNA extraction of *W. magna* c2c maky was carried out using the RNeasy Mini Kit (Qiagen, Hilden, Germany). The QuantiFluor RNA sample system (Promega) was used to quantify total RNA from each sample. Total RNA was qualified using Nano RNA chip on BioAnalyser 2100 (Agilent Technologies Inc.). A poly A capture was performed to purify mRNA from the total RNA. Libraries were then generated using NextFlex rapid directional RNAseq sample prep (Bioo Scientific Corporation). Libraries were quantified using dsDNA HS Assay on Quantus Fluorometer and qualified on BioAnalyser 2100 from Agilent using HS DNA chip. Sizes of fragments in the library were about 380 bp, therefore compatible with cluster generation. Paired-end sequencing with 75 bp read length was performed on NextSeq. 500 Mid Output flow cell lines from Illumina generating 130 million reads. The sequencing of two RNA samples generated 216.93 million of raw reads, 77.4% of which met the Illumina filtering criteria.

### Genome annotation and analysis of taxonomical distribution

The quality of raw data from DNA and RNA sequencing was controlled using the FastQC software (https://www.bioinformatics.babraham.ac.uk/projects/fastqc/). Raw data were trimmed with the Trimmomatic software^54^. Indeed, the reads with low quality and adapters were removed and only the reads with average quality above 28 were selected. All trimmed reads of DNA were *de novo* assembled using CLC Genomics Workbench v7.51 (https://www.qiagenbioinformatics.com/products/clc-genomics-workbench/). The 64-word size and 100 bubble size parameters were used. The contig with size under 900 bp were removed. The assembly was improved with GapFiller^55^. To screen the presence of the contaminant in assembly, BlobTools^56^ was used. The software used guanine-cytosine content of sequences, read coverage in sequencing libraries and taxonomy of sequence similarity matches to visualize the quality control and taxonomic partitioning of genome datasets. Protein coding genes prediction was performed using BRAKER1, which applies an *ab-initio* approach taking into account the RNAseq data^57^. First, BRAKER1 uses GeneMark-ET^58^ to perform iterative training and generate the initial gene structures. In a second step, AUGUSTUS uses predicted genes for training and then integrates RNA-Seq read information into final gene predictions^59,60^. Protein annotation was performed on NCBI GenBank non-redundant protein sequence database (nr) using BLAST protein with an e-value of 1e-03 as significance thresholds. Functional classification of gene families (COG ID and letters) was obtained using EggNOG against the COG database^61^. Taxonomical origins were determined using MEGAN6^62^.

### Comparative genomic analyses

To identify orthologs between different amoebas within the family *Vahlkampfiidae*, we used Proteinortho v5^63^ with 60% coverage and 50% amino acid identity, and an e-value of 1e-4 as significance thresholds. The clustering was obtained from the predicted proteins using AUGUSTUS program for *W. magna*, *N. fowleri* (GCA_000499105.1), *N. gruberi* (GCA_000004985.1) and *N. lovaniensis* (GCA_003324165.1). The representation of core and pangenome of *Vahlkampfiidae* genes was performed using the Bioinformatics and Evolutionary Genomics online platform (http://bioinformatics.psb.ugent.be/webtools/Venn/). Furthermore, a pangenomic tree was generated using the GET_HOMOLOGUES package^64^ with the standard parameters. Next, phylogenetic analysis based on the 18S rRNA gene was performed. The 18 s rRNA gene of *W. magna* c2c maky was detected by BLASTn comparison between the amoebal genome assembly and the published 18 s rRNA sequences of other *W. magna* strains (AY266315, KC164225.1). Homologs were searched using BLASTn against nucleotide collection (nr/nt). Multiple sequence alignment was carried out using the Clustal W software^65^. Finally, a phylogenetic analysis of these nucleotide sequences was performed using the MEGA version 7^66^ and the maximum likelihood (ML) algorithm, with 1,000 bootstrap replicates.

### Putative horizontal gene transfers

The *W. magna* genes best matching with genes from amoeba-resistant microorganisms have been identified and for each protein sequence, a BLASTp search was performed against non-redundant protein sequences database (nr), with e-value cutoff 1e-03. Phylogenetic analyses were carried out to confirm the potential horizontal transfers for the genes exhibiting the best matching with amoeba-resistant microorganism homologs. Protein sequences were aligned using MUSCLE^67^. Phylogenetic trees were obtained using the FastTree software^68^ and the maximum likelihood method with Jones-Taylor-Thornton (JTT) model on MEGA 7.0.25 software. Phylogenetic trees were analyzed using iTOL v3 online^69^.

### Analysis of potential virulence related genes

Genes of bacterial human pathogens detected in the *Willaertia* genome were identified as best hits and listed. Hypothetical proteins were excluded from the protein sequence list. The protein sequences were compared by BLASTp against available protein sequences downloaded from the Virulence Factor of Pathogenic Bacteria database (VFDB)^70^. We also investigated proteins for which the best hit was from the human pathogen *N. fowleri*. The proteins of *W. magna* that had a significant hit and that were related to virulence were used as queries to search against the nr database. Phylogenetic analyses were conducted for various virulence related proteins. Amino acid sequence alignments were performed using Clustal W and phylogeny reconstructions were carried out using the maximum likelihood method on MEGA version 7 software. The visualization of the phylogenetic trees was finally performed using the iTOL v3 online tool.

### Cell preparation for cytotoxicity assay

*W. magna* was grown and prepared as described above in order to obtain 5.10^5^ cells/ml in PAS medium for the cytotoxicity tests. *B. mandrillaris* (ATCC50209) was grown on monolayer of African green monkey kidney cells (ATCC CRL1586: Vero cells). Briefly, *B. mandrillaris* was inoculated in 10 mL RPMI 1640 with 5% fetal calf serum on a Vero cells grown in culture flasks. The trophozoites were harvested after 72 hours, centrifuged and suspended in PAS medium. Quantification was performed using KOVA® slide counting method to obtain 5.10^5^ cells/ml which were used for subsequent assays.

Amoeba lysates (*B. mandrillaris* and *W. magna*) were obtained by heat shocks where trophozoites (5.10^5^ cells/mL) were processed during 1 minute in liquid nitrogen followed by 1 minute at 45 °C. This process was repeated three times.

The human microvascular endothelial cell (ECs) line HMEC-1 was grown on culture flask in RPMI 1640 containing 10% heat-inactivated fetal bovine serum, penicillin (100 U/mL) and streptomycin (100 mg/mL). ECs were harvested using trypsin/EDTA after 15 minutes of incubation with Hank’s Balanced Salt Solution at room temperature.

### ICELLigence assay

Cell adhesion, proliferation and survival were monitored in real-time process through the iCELLigence™ Real-Time Cell Analysis (RTCA) systems (ACEA Bioscience, Inc., San Diego, CA) by measuring cell-to-electrode responses of the cells seeded in eight-well E-plates with the integrated microelectronic sensor arrays. The iCELLigence™ measured the impedance or cell index factor. ECs were seeded at a density of 16,000 cells per well in a total volume of 400 μl of endothelial cells medium and were monitored real-time for 3 days. The ECs herein are the target cells, which are the cells responding to effector and whose impedance has been measured. The effectors in our case are the staurosporine as a positive control for cytotoxicity and cell death, the PAS as a negative control, the *W. magna*, and the *B. mandrillaris* as candidates for the assay, which are in the L16 inserts (ACEA Bioscience, Inc., San Diego, CA). These cells are the effector cells. The impedance of these cells was not measured since they were cultured in the E-Plate L16 insert device.

### Cytotoxicity assay rolling

The E-Plate inserts and E-Plate L8 devices are incubated separately for 30 minutes prior to assembly for the assay. This allows uniform cell seeding on the E-Plate L8 (ACEA Bioscience, Inc., San Diego, CA) as well as on the E-Plate insert membrane. We then incubated at 37 °C and monitored the E-Plate L8 device for five hours as the target cells adhered and proliferated. The E-Plate Inserts (in the receiver plate) were also introduced in the same incubator in order to have the target cells and effectors cells under the same conditions and environment. After 5 hours of initial proliferation, the L16 insert was gently inserted and the cells were monitored every 15 min for 30 to 48 hours. The L16 insert has pores (0.20 µm) to allow only nanoparticles or potential toxins to enter wells containing ECs targets. Each insert contained 120 µl of samples. These samples are composed of either *W. magna* trophozoites forms (60,000 cells/insert) or *B. mandrillaris* trophozoites forms (60,000 cells/insert) or *W. magna* lysate or *B. mandrillaris* lysate. The negative control was the PAS medium and staurosporine (0.4UL, sigma) was used as positive control. The manipulation was carried out in triplicate.

## Supplementary information

Supplementary data

Table S1

Table S2

Tables S3

Tables S4

Tables S5

## Data Availability

The genome is deposited in EBI-EMBL under the bioproject number PRJEB30797. The datasets generated during the current study are available at https://www.mediterranee-infection.com/acces-ressources/donnees-pour-articles/willaertia-magna-c2c-maky/. The link provides direct access to the genome assembly data of *W. magna*. After downloading the file, it has to be unzip in order to read the data in Fasta format.
