# Supplementary material for: Investigation of potential pathogenicity of Willaertia magna by investigating the transfer of bacteria pathogenicity genes into its genome
Source: Sci Rep. 2019 Dec 4;9:18318. doi: 10.1038/s41598-019-54580-6 (PMC6892926; doi:10.1038/s41598-019-54580-6)

Title: Investigation of potential pathogenicity of *Willaertia magna* by investigating the transfer of bacteria pathogenicity genes into its genome

Authors: Issam Hasni[1,2], Nisrine Chelkha [1], Emeline Baptiste[1], Mouh Rayane Mameri[2], Joel Lachuer[3,4,5], Fabrice Plasson[2], Philippe Colson [1] and Bernard La Scola[1]*

Supplementary table legends:

Supplementary table S 1: Functional analysis of *Willaertia magna* gene content.

Supplementary table S 2: List of *W. magna* protein sequences had BLASTp best hits with Amoeba resistant microorganisms including amoeba resistant bacteria and giant viruses.

Supplementary table S 3: List of *W. magna* protein sequences with known function had BLASTp best hits with bacteria, mostly living in the environment, and identified as potentially pathogenic for humans.

Supplementary table S 4: List of *W. magna* protein sequences had BLASTp best hits with *Naegleria Fowleri*.

Supplementary table S 5: BLASTp results of protein sequences (Actin, *Hsp70*, membrane protein and *Nf314*) belonging at *Naegleria fowleri*.

Supplementary data

Figure S1: Blobplot of *W. magna* c2c maky assembly. Sequences in the assembly are represented by circles, with diameter proportional to sequence length and colored by taxonomic annotation. Circles are positioned on the X-axis based on their GC proportion and on the Y-axis on the coverage.

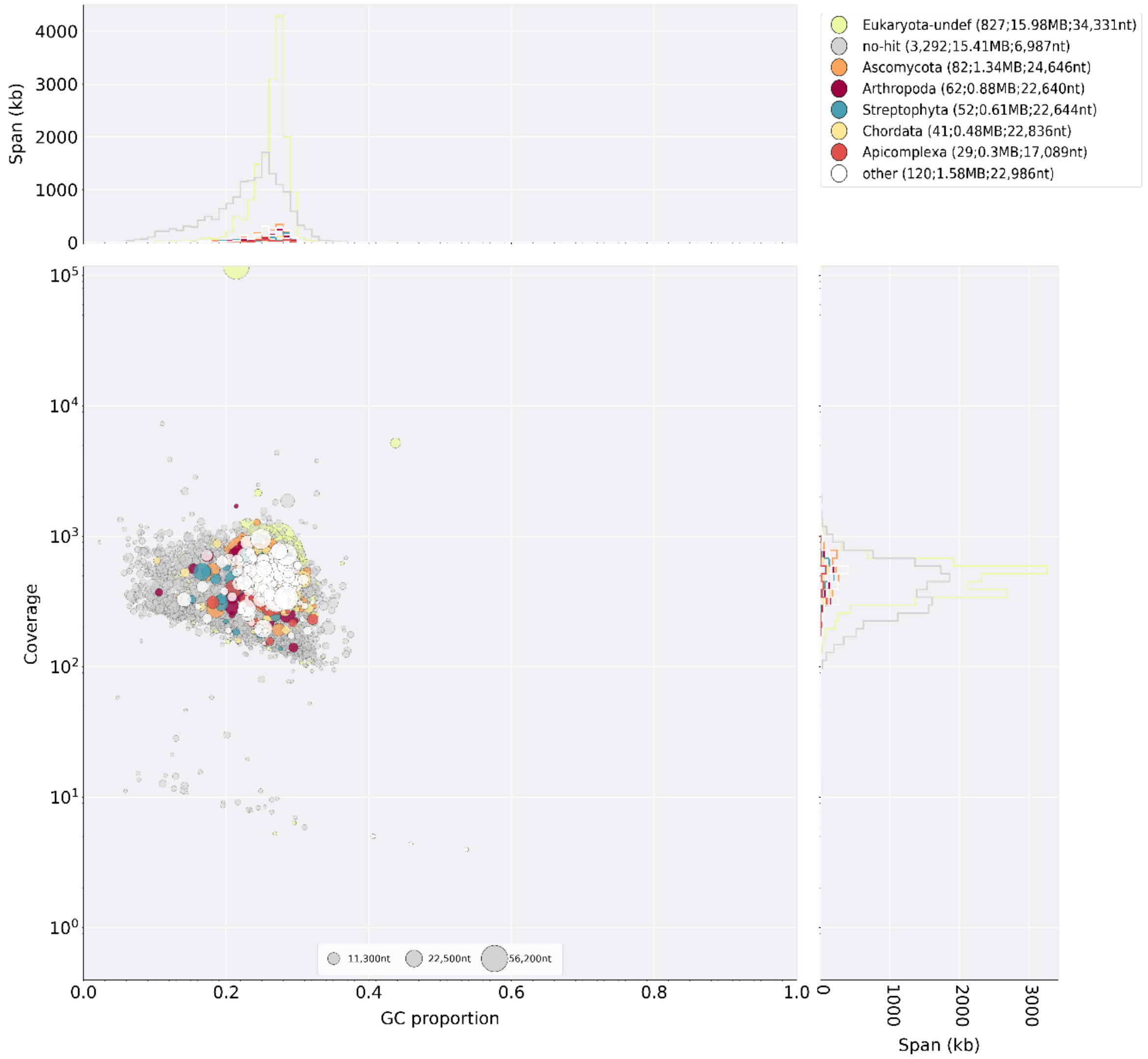

Figure S2: Phylogenetic analysis of amoebas of *Heterolobosea* and *Amoebozoa* clade. The phylogenetic tree is based on amoeba partial available SSU rRNA sequences of amoebas. GenBank Accession numbers are indicated in parentheses. The sequences were aligned by ClustalW and tree was performed using Jukes-Cantor model on MEGA 7.0.25 software. Numbers at the nodes are percentages of bootstrap values obtained by repeating the analysis 1,000 times to generate a consensus tree; only values ≥ 0,7 were displayed. The scale bar indicates a 10% nucleotide sequence divergence.

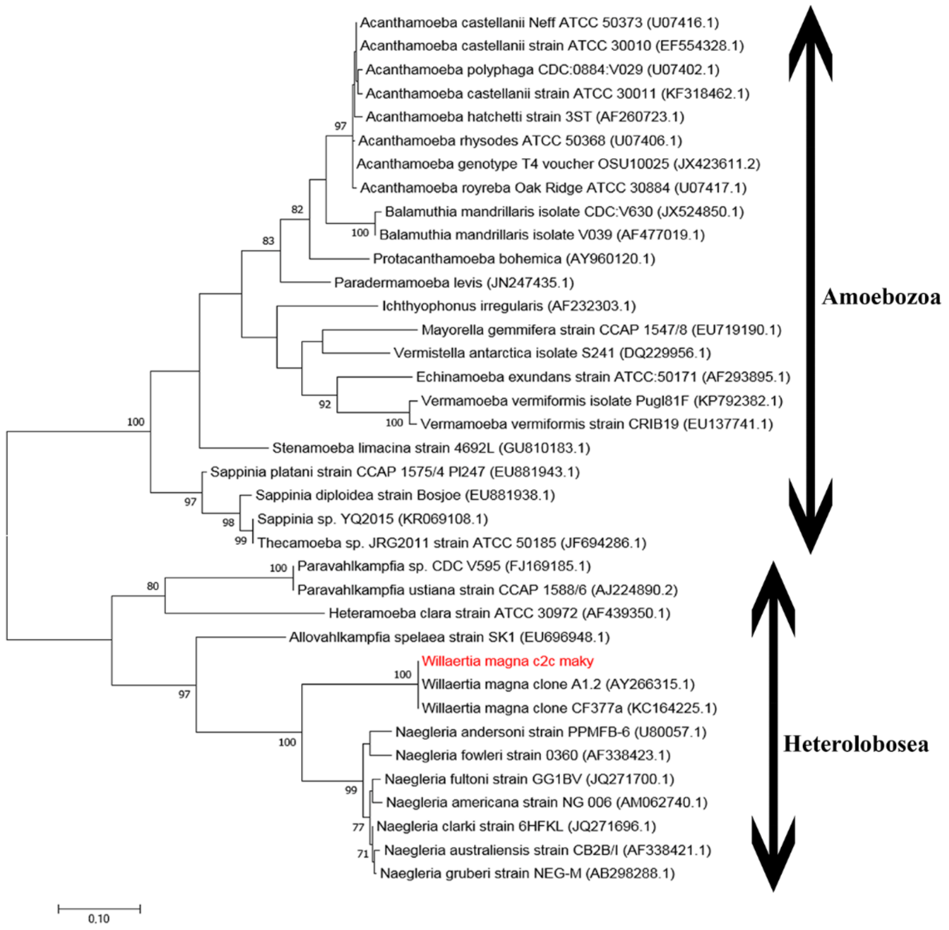

Figure S3: Representation of COG functional categories for the amoebas of *Naegleria* species. a) COG of *Naegleria gruberi*, b) COG of *Naegleria fowleri*, c) COG of *Naegleria lovaniensis*, d) comparison of the percentage of genes related to cytoskeletons.

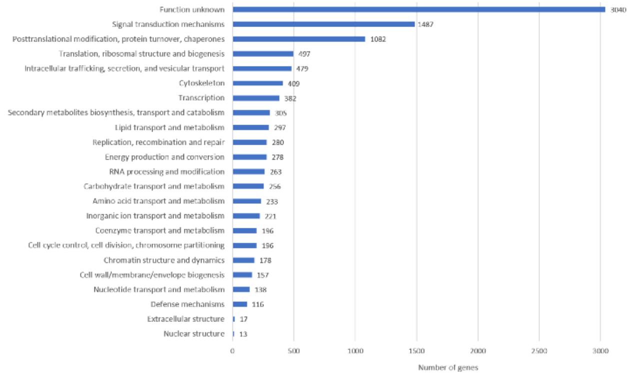

(a)

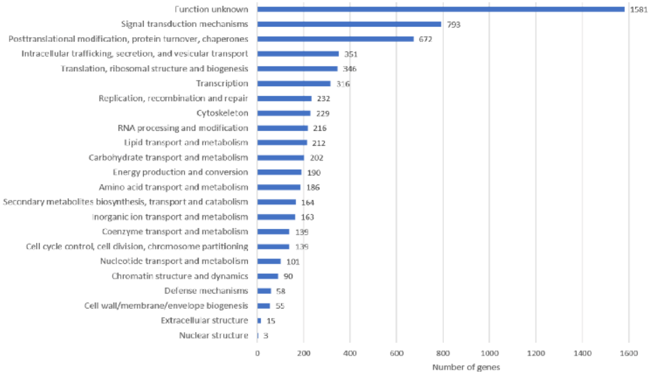

(b)

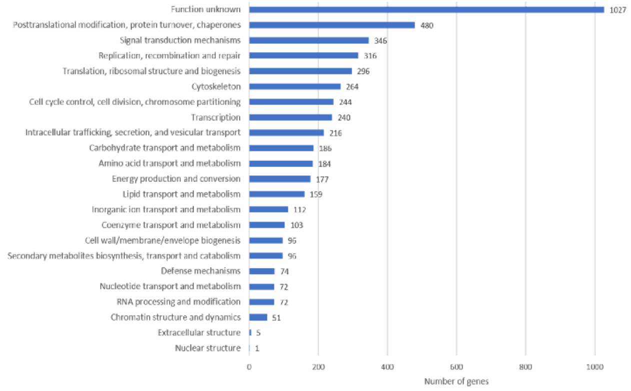

(c)

	W. magna	*N. gruberi*	*N. fowleri*	*N. lovaniensis*
Percentage of cytoskeleton genes	3,40%	2.34%	2.34%	2.30%

(d)

Figure S4: Representation of the *W. magna* genes related to metabolism. The analysis was performed by comparison of *W.magna* protein sequences with KEGG database (www.kegg.jp/kegg/kegg1.html) (Kanehisa, M. & Goto, S. KEGG: Kyoto Encyclopedia of Genes and Genomes. *Nucleic Acids Res.* **28**, 27-30 (2000)).

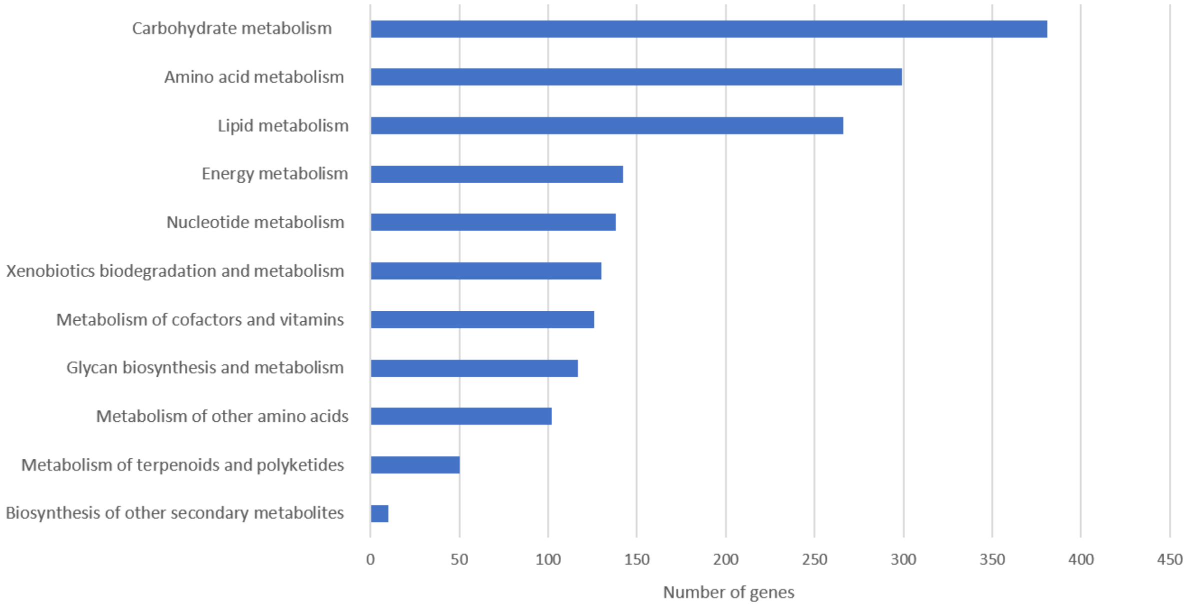

Figure S5: Metabolic pathway of the terpenoid biosynthesis in which a *W. magna* gene (isopentenyl-diphosphate Delta-isomerase; 5.3.3.2) is involved (from KEGG database; www.kegg.jp/kegg/kegg1.html) (Kanehisa, M. & Goto, S. KEGG: Kyoto Encyclopedia of Genes and Genomes. *Nucleic Acids Res.* **28**, 27-30 (2000)).

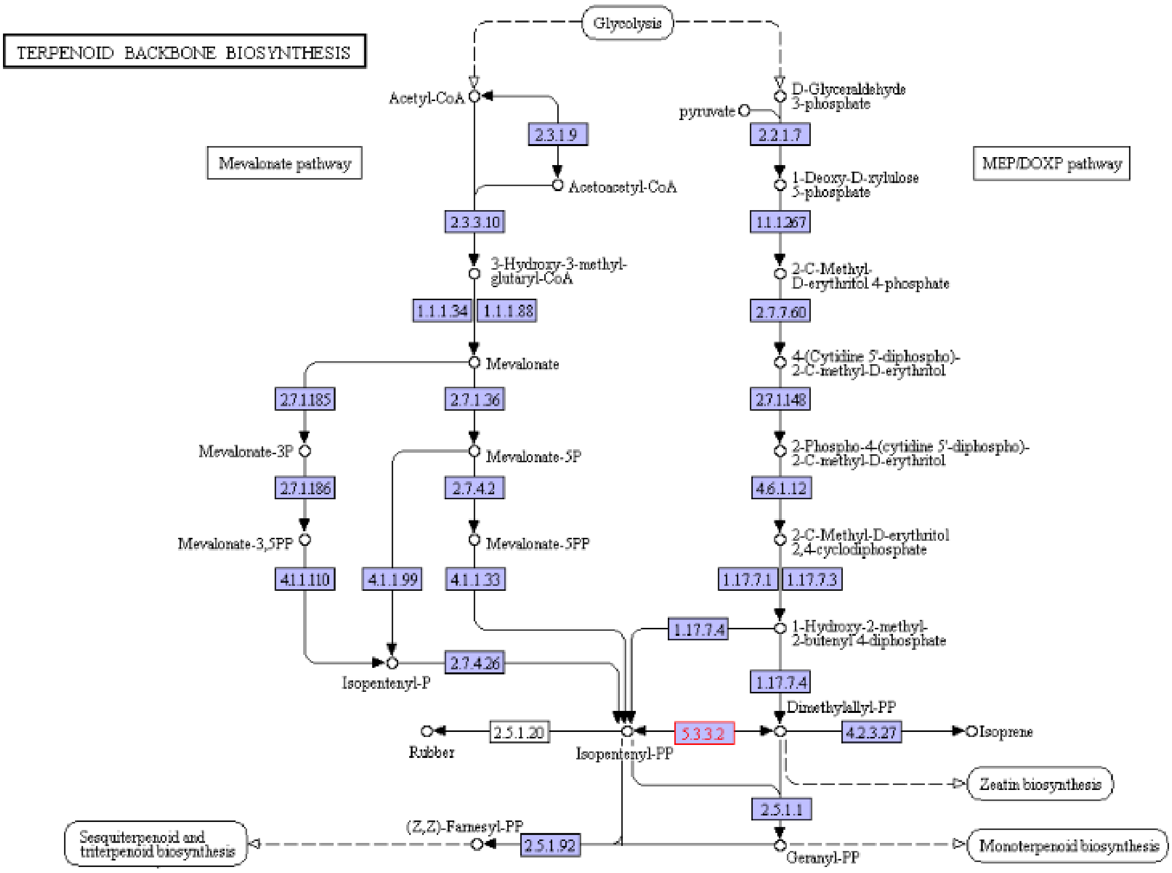

Figure S6: Metabolic pathway of the terpenoid biosynthesis in which a *W. magna* gene (diphosphomevalonate decarboxylase; 4.1.1.33) is involved (from KEGG database; www.kegg.jp/kegg/kegg1.html) (Kanehisa, M. & Goto, S. KEGG: Kyoto Encyclopedia of Genes and Genomes. *Nucleic Acids Res.* **28**, 27-30 (2000)).

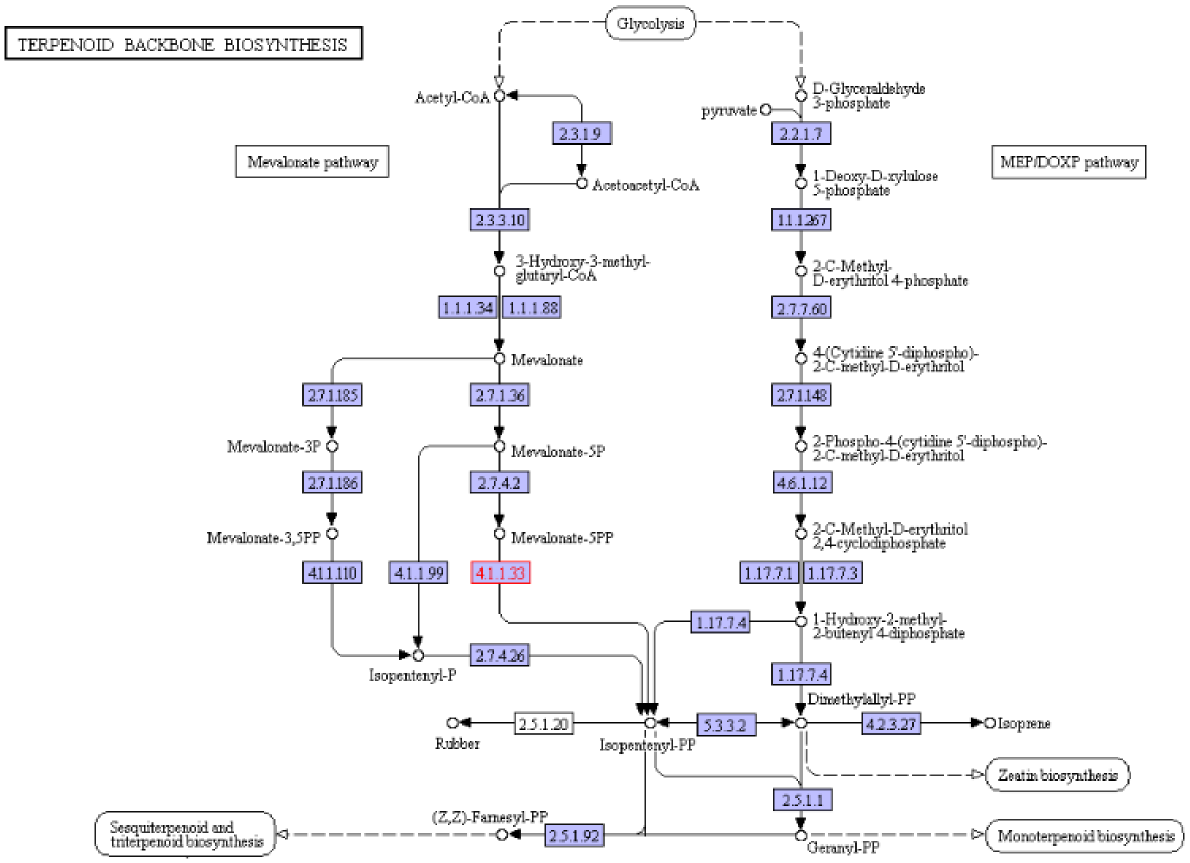

Figure S7: Metabolic pathway of the terpenoid biosynthesis in which the *W. magna* gene (farnesyl-diphosphate farnesyltransferase; 2.5.1.21) is involved (from KEGG database; www.kegg.jp/kegg/kegg1.html) (Kanehisa, M. & Goto, S. KEGG: Kyoto Encyclopedia of Genes and Genomes. *Nucleic Acids Res.* **28**, 27-30 (2000)).

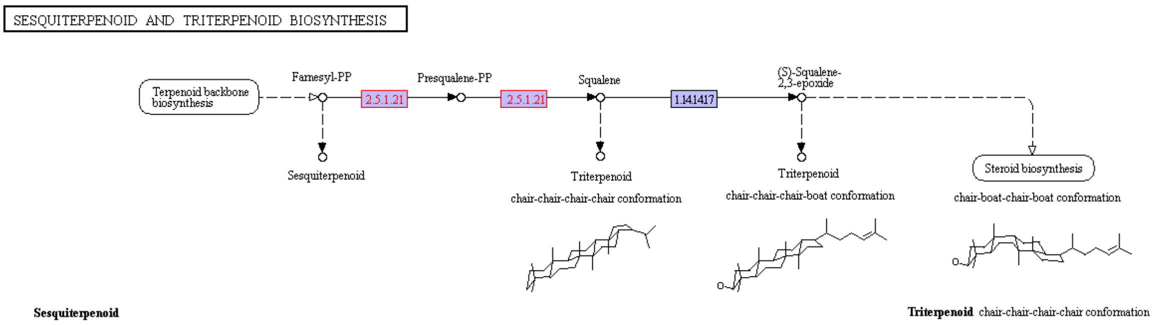

Figure S8: Metabolic pathway of the penicillin biosynthesis in which a *W. magna* gene (penicillin amidase; 3.5.1.11) is involved (from KEGG database; www.kegg.jp/kegg/kegg1.html) (Kanehisa, M. & Goto, S. KEGG: Kyoto Encyclopedia of Genes and Genomes. *Nucleic Acids Res.* **28**, 27-30 (2000)).

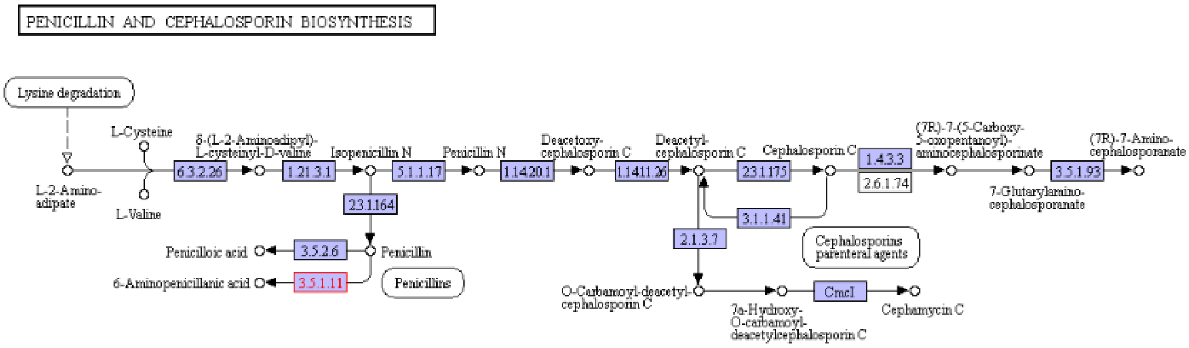

Figure S9: Representation of COG functional categories of the core genome.

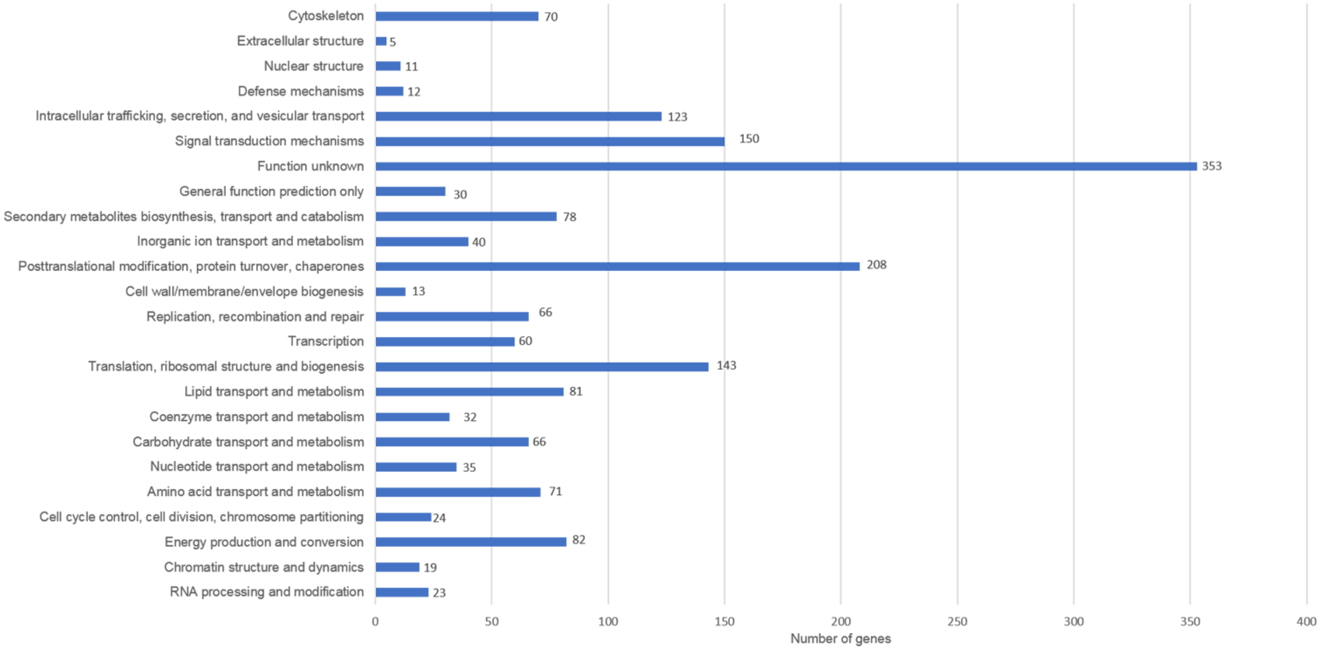

Figure S10: Representation of COG functional categories of the unique genes of *W. magna* c2c.

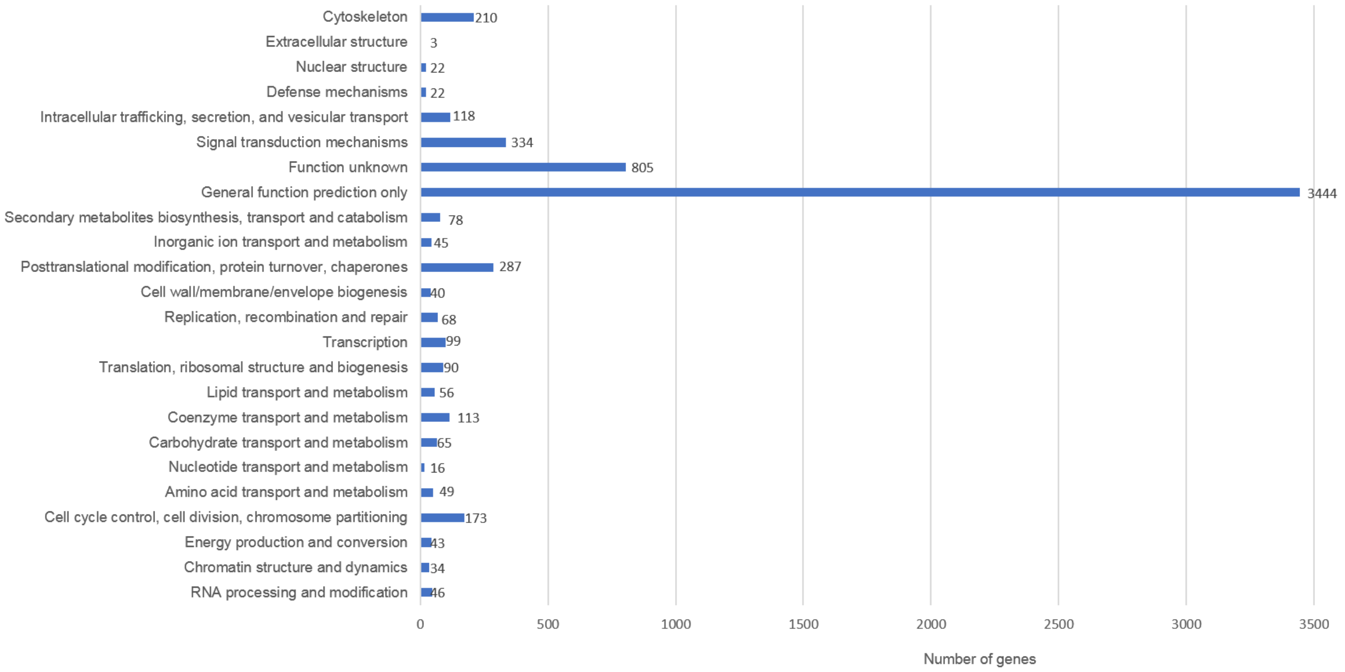

Figure S 11: Hierachical clustering based on pangenome analysis of *W. magna* c2c maky, *N. gruberi*, *N. fowleri* and *N. lovaniensis*. Pangenome and hierachical clustering were performed by the get_Homologues software. The representation is based on the absence/presence patterns of homologous genes for the different organisms used in the study. In black: *W. magna* c2c maky. In red: amoebas of the genus *Naegleria*.

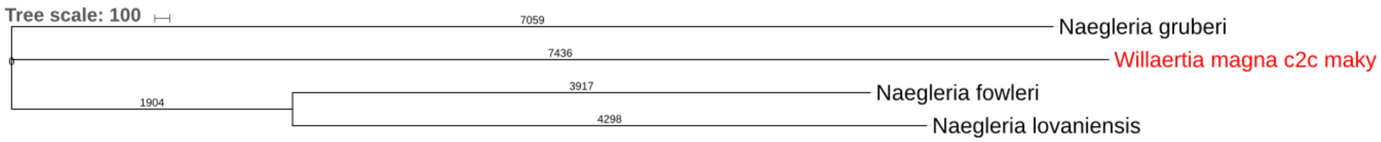

Figure S12: Representation of COG functional categories of the genes shared between *W. magna* c2c maky and amoeba-resisting microorganisms.

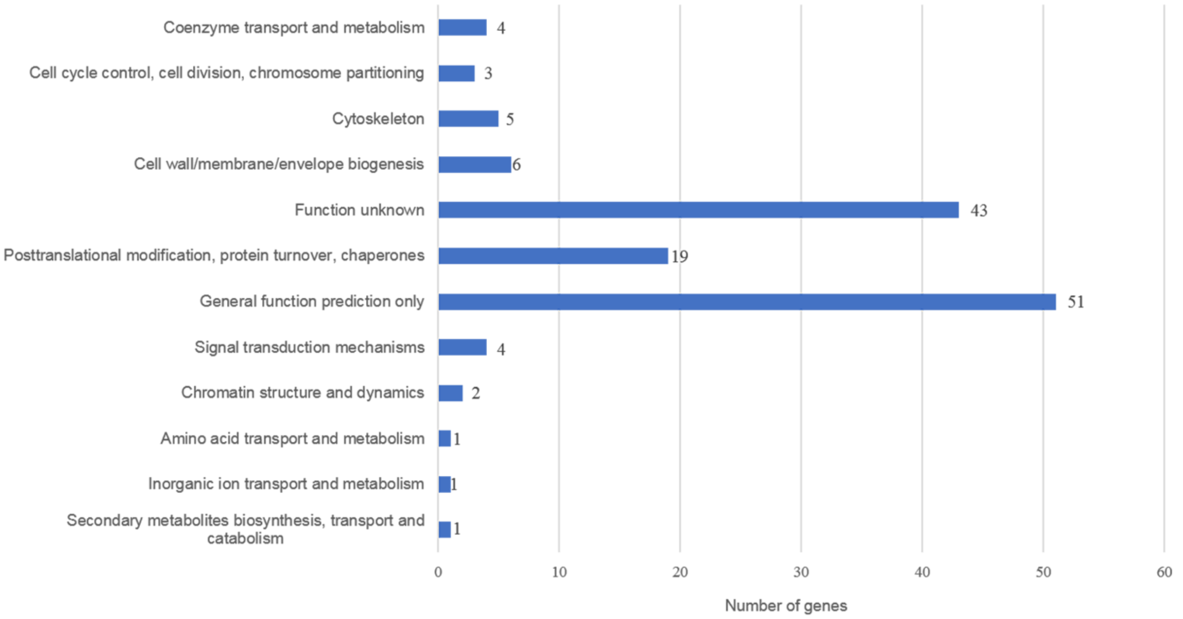

Figure S13: Representation of horizontal transfers analysis. Phylogenetic trees for two *W. magna* c2c maky proteins (A and B) whose encoding genes were putatively transferred to giant viruses. The sense of the sequence transfers was inferred from the results of BLASTp

best hits searches and from the topology of the trees. In red: *W. magna* c2c maky gene; in green: the best viral homolog; in blue: homologs from other giant viruses; in black: homologs from other organisms.

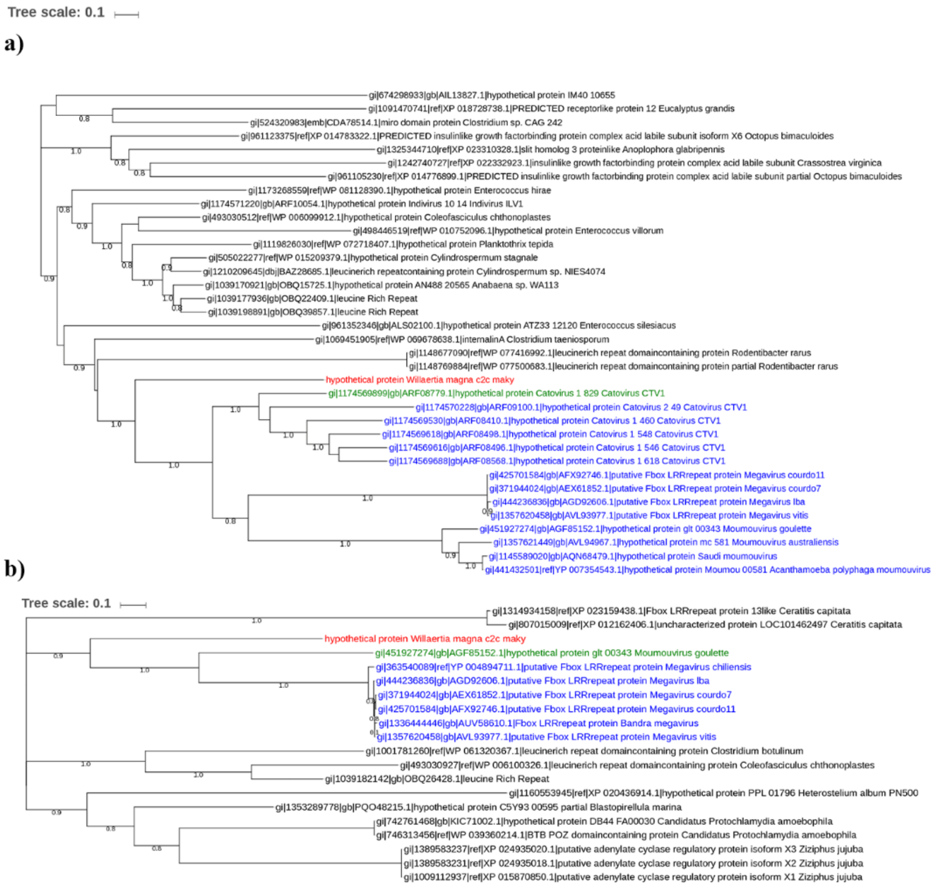

Figure S14: Phylogenetic tree based on amoeba homologous of *W. magna* c2c (*Hsp70*: A, actin: B, membrane protein C). GenBank Accession numbers are indicated in parentheses.

The sequences were aligned by ClustalW and trees were performed using Jukes-Cantor model on MEGA 7.0.25 software. Numbers at the nodes are percentages of bootstrap values obtained by repeating the analysis 100 times to generate a majority consensus tree. Only values ≥ 0,7 were displayed. The *Hsp70* (A), actin (B), membrane protein (C) of *W. magna* c2c are indicated in green; in blue: homologs from non-pathogenic amoebas and red: homologs from pathogenic amoeba (*N. fowleri*); in black: homologs from other organisms.

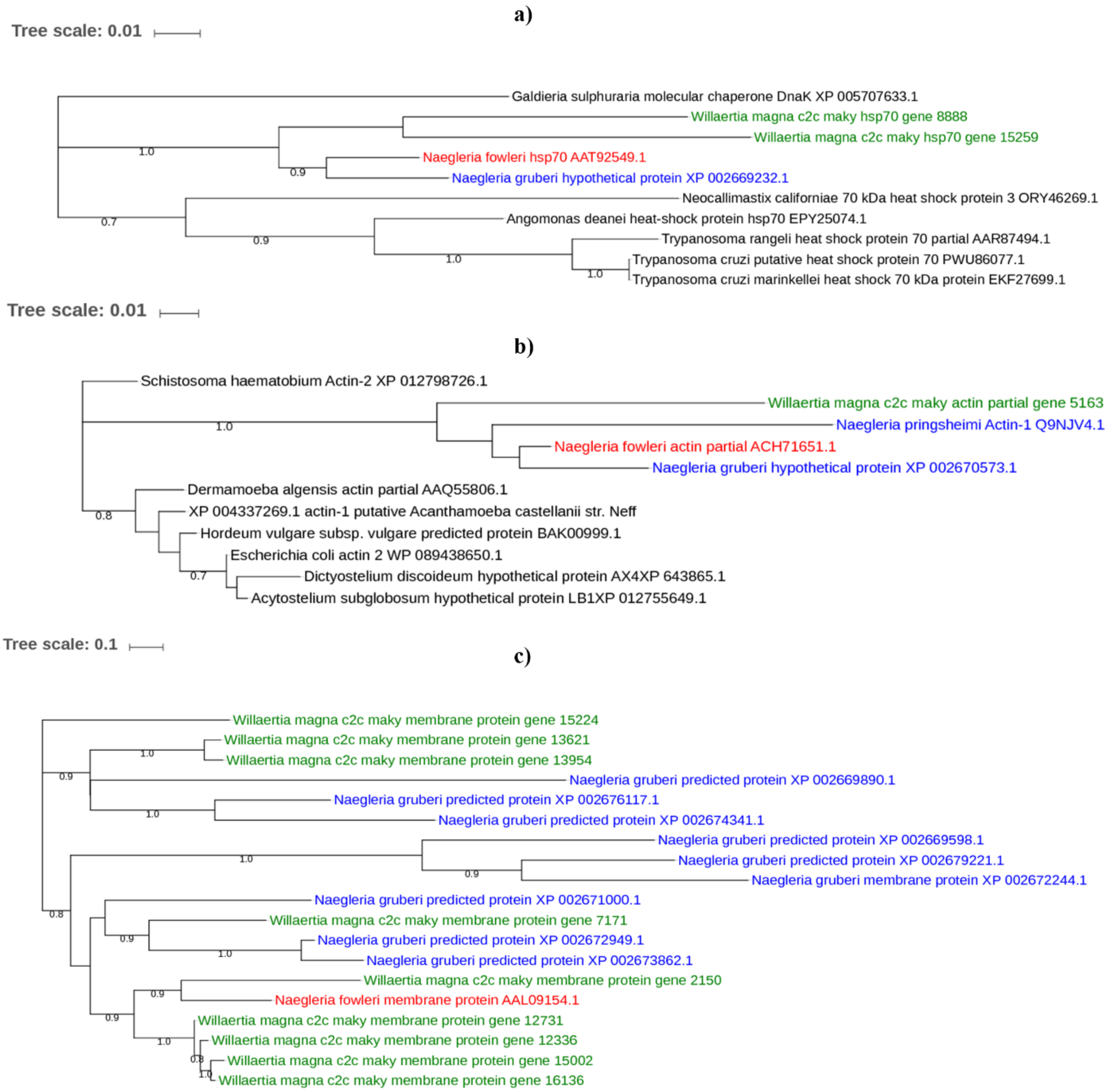

Supplement: Supplementary file 1 — Supplementary data [file 41598_2019_54580_MOESM1_ESM.pdf]
